# Plant chromosome polytenization contributes to suppression of root growth in high polyploids

**DOI:** 10.1093/jxb/erae288

**Published:** 2024-07-05

**Authors:** Suzuka Kikuchi, Takuya Sakamoto, Sachihiro Matsunaga, Munetaka Sugiyama, Akitoshi Iwamoto

**Affiliations:** Faculty of Advanced Science and Technology, Kumamoto University, 2-39-1 Kurokami, Chuou-ku, Kumamoto-shi, Kumamoto, 860-8555, Japan; Faculty of Science, Kanagawa University, 3-27-1 Rokkakubashi, Kanagawa-ku, Yokohama-shi, Kanagawa, 221-8686, Japan; Faculty of Science and Technology, Tokyo University of Science, 2641 Yamazaki, Noda-shi, Chiba, 278-8510, Japan; Graduate School of Frontier Sciences, The University of Tokyo, 5-1-5 Kashiwanoha, Kashiwa-shi, Chiba, 277-8561, Japan; Graduate School of Science, The University of Tokyo, 7-3-1 Hongo, Bunkyo-ku, Tokyo, 113-0033, Japan; Faculty of Science, Kanagawa University, 3-27-1 Rokkakubashi, Kanagawa-ku, Yokohama-shi, Kanagawa, 221-8686, Japan; University of Antwerp, Belgium

**Keywords:** *Arabidopsis thaliana*, autopolyploid, chromosome polytenization, endoreduplication, high polyploid, kinematic analysis, root growth, whole-mount FISH

## Abstract

Autopolyploidization, which refers to a polyploidization via genome duplication without hybridization, promotes growth in autotetraploids, but suppresses growth in high polyploids (autohexaploids or auto-octoploids). The mechanism underlying this growth suppression (i.e. ‘high-ploidy syndrome’) has not been comprehensively characterized. In this study, we conducted a kinematic analysis of the root apical meristem cells in *Arabidopsis thaliana* autopolyploids (diploid, tetraploid, hexaploid, and octoploid) to determine the effects of the progression of genome duplication on root growth. The results of the root growth analysis showed that tetraploidization increases the cell volume, but decreases cell proliferation. However, cell proliferation and volume growth are suppressed in high polyploids. Whole-mount fluorescence *in situ* hybridization analysis revealed extensive chromosome polytenization in the region where cell proliferation does not usually occur in the roots of high polyploids, which is likely to be at least partly correlated with the suppression of endoreduplication. The study findings indicate that chromosome polytenization is important for the suppressed growth of high polyploids.

## Teaser

This study has elucidated the relationship between chromosome polytenization and growth changes in autopolyploid series of *Arabidopsis thaliana* , including high polyploids.

## Introduction

Polyploidy, which refers to the presence of three or more chromosomal sets in an organism, occurs repeatedly across diverse taxonomic groups of eukaryotes. Previous reports described the polyploidy in a variety of eukaryotic species, such as fungi (Albertin and Marullo, 2012; Todd *et al.*, 2017), arthropods (Otto and Whitton, 2000; Li *et al.*, 2018), mollusks (Otto and Whitton, 2000; Gregory and Mable, 2005), fishes (Le Comber and Smith, 2004; Mable *et al.*, 2011), amphibians (Schmid *et al.*, 2015; Session *et al.*, 2016), and land plants (Jiao *et al.*, 2011; Scarpino *et al.*, 2014), but only a few species of birds and mammals (Gallardo *et al.*, 1999; Otto and Whitton, 2000). Polyploidization is an important phenomenon in a wide range of research fields, such as physiology, evolutionary biology, and ecology, because it is generally believed to contribute to the increase in gene expression caused by the dosage effect and changes in the gene expression regulatory network, which may lead to adaptive evolution (te Beest *et al.*, 2012; Wertheim *et al.*, 2013). The frequency of polyploidization events varies among species; they are relatively rare in fungi and animals, but common in land plants. Therefore, polyploidization is a major driver of plant speciation (Soltis *et al.*, 2003; Jiao *et al.*, 2011; Scarpino *et al.*, 2014; Van de Peer *et al.*, 2021).

The two types of polyploids in plants are autopolyploids, whose chromosome sets come from the same species, and allopolyploids, which are derived from interspecific hybridization (Kihara and Ono, 1926). Historically, allopolyploids were considered to be more abundant in nature and important for plant speciation, which may explain why they have received more attention than autopolyploids (Spoelhof *et al.*, 2017). However, clarifying the effects of autopolyploidization on plant growth is important because it can provide the basis for analyses of the effects of allopolyploidization. Allopolyploids are the result of hybridization and genome duplication (Soltis and Soltis, 2009), whereas autopolyploidization only involves the genome duplication process. Thus, studying the effects of autopolyploidization may provide useful insights for research on the effects of allopolyploidization.

Several previous studies examined artificially generated *Arabidopsis thaliana* autopolyploids to determine the effects of autopolyploidization on plants. The results of these studies indicated that the autopolyploidization of tetraploids promotes the growth of several organs. Moreover, tetraploids also reportedly have larger and fewer cells than diploids [e.g. sepal cells (Robinson *et al.*, 2018), pavement cells (Corneillie *et al.*, 2019), and root cortical cells (Iwamoto *et al.*, 2006)]. In contrast, the suppressed growth of high polyploids (e.g. hexaploids and octoploids) has been referred to as ‘high-ploidy syndrome’ (Tsukaya, 2008). Similar to tetraploids, high polyploids have larger and fewer cells than diploids (Tsukaya, 2008; Robinson *et al.*, 2018; Corneillie *et al.*, 2019); however, their growth is severely delayed and they exhibit dwarfism (compared with diploids and tetraploids) (Kikuchi and Iwamoto, 2020). Although high-ploidy syndrome has been characterized to some extent, it is still unclear how it suppresses plant growth, although cell cycle delays might be involved (Comai, 2005). Whether cell proliferation and/or cell volume increase are repressed in specific organ parts of high polyploids remains unknown.

The root is a good model for analyzing plant organ growth because it has a relatively simple structure (Dolan *et al.*, 1993) and grows almost one-dimensionally (Erickson, 1976) with spatially divided growth zones (i.e. cell proliferation, transition, elongation, and mature zones) (Verbelen *et al.*, 2006). In previous studies, kinematic analyses of *A. thaliana* root apical meristem cells were performed to quantitatively elucidate the spatial profile of specific growth parameters (Beemster and Baskin, 1998; West *et al.*, 2004; Iwamoto *et al.*, 2006). For example, Iwamoto *et al.* (2006) characterized the root growth parameters of autotetraploid and diploid *A. thaliana* and showed that the autopolyploidization that generates tetraploids leads to an increase in the cell volume and the suppression of cell proliferation (Iwamoto *et al.*, 2006). In the current study, we established a series of *A. thaliana* autopolyploids, including high polyploids, and performed a kinematic analysis of cells to determine the effect of the progression of autopolyploidization on the spatial profile of individual root growth parameters.

We also elucidated the mechanism underlying the change in organ growth due to autopolyploidization. The altered growth of autopolyploids is caused by the changes to transcript levels associated with genome duplication events. Song *et al.* (2020) reported that the total transcript abundance increased in autotetraploid *A. thaliana*, but it was less than double that of its diploid ancestors, suggesting that autopolyploidization may increase gene expression, but the increase is not proportional to the genome size. In general, chromosome structural changes are associated with changes in gene expression (Jarillo *et al.*, 2009; Song *et al.*, 2021). Therefore, autopolyploidization-induced modifications to chromosome structures may modulate gene expression, which should be considered when examining the mechanism underlying the growth changes due to autopolyploidization. Chromosome polytenization in the leaf cells of *A. thaliana* autopolyploids (Breuer *et al.*, 2007; Kikuchi and Iwamoto, 2020) was confirmed via a fluorescence *in situ* hybridization (FISH) analysis with a centromeric DNA probe. In these studies, they and we reported the smaller number of centromere signals than the number of chromosomes, which suggested the adhesion or contact of chromosomes, at least in the centromeric region. We defined the adhesion or contact of chromosomes as chromosome polytenization in this study. The decrease in the number of centromere signals enables us to estimate the degree of chromosome polytenization. The chromosome polytenization in autopolyploids may affect gene expression as well as cell proliferation because the polytenization influences chromosome segregation.

In this study, we used a centromeric DNA probe to perform a whole-mount FISH (WM-FISH) analysis of the whole root apical meristems of autopolyploids. The objective of this study was to clarify the spatial profile of the degree of chromosome polytenization in the root apical meristems of *A. thaliana* autopolyploids. We subsequently compared the results of the WM-FISH analysis with the results of a root growth analysis to elucidate the relationship between growth traits and chromosome dynamics. This study provides insights into the mechanisms mediating the growth changes due to the progression of autopolyploidization.

## Materials and methods

### Production of synthetic *Arabidopsis thaliana* autopolyploids

Synthetic *A. thaliana* (L.) Heynh. autopolyploids (tetraploid, hexaploid, and octoploid) were produced by treating diploid (Columbia-0) seedlings with colchicine (Kikuchi and Iwamoto, 2021). The ploidy level was determined by a flow cytometry analysis. The ploidy level of the generated autopolyploid lines was confirmed for at least three generations after the colchicine treatment before the autopolyploids were used in this study. Two independent lines for each autopolyploid (tetraploid-1 and -2; hexaploid-1 and -2; and octoploid-1 and -2) were established. All autopolyploid lines were used for the kinematic analysis, whereas tetraploid-1, hexaploid-1, and octoploid-1 were used for the WM-FISH analysis.

### Flow cytometry analysis

Leaf samples for the flow cytometry analysis were collected from seedlings at 30–40 d after sowing. The ploidy level of each line was verified using a flow cytometer (CyFlow Ploidy Analyzer PA; Sysmex Partec GmbH, Münster, Germany) and the previously described chopping method (Johnston *et al.*, 1999).

### Plant materials and growth conditions

The seeds of *A. thaliana* (Columbia-0) autopolyploids (diploid, tetraploid, hexaploid, and octoploid) were surface-sterilized in a 20% sodium hypochlorite solution containing 0.5% Triton X-100. The seeds were sown in each growth medium in plates, which were then incubated vertically for 8 d (for the kinematic analysis and WM-FISH analysis) or 10 d (for measuring the root elongation rate) under constant conditions (22 °C and continuous light at 90 μmol m^−2^ s^−1^). The growth media used in this study contained the following common components (per liter): 10 g of sucrose, 0.5 g of MES, 10 ml of 100× Murashige and Skoog (MS) vitamins (10 mg l^–1^ thiamine HCl, 50 mg l^–1^ pyridoxine HCl, 50 mg l^–1^ nicotinic acid, 10 g l^–1^ myo-inositol, and 200 mg l^–1^ glycine), and half-strength (2.3 g) MS Plant Salt Mixture (Wako Pure Chemical Industries, Osaka, Japan). The solid medium contained 0.8% (w/v) gellan gum (Lot #SLBV6512, G1910-250G; Sigma Life Science, St. Louis, MO, USA).

### Measurement of the primary root growth

The position of the root apical meristem of each seedling was recorded daily for 4–10 d after sowing to determine the days when the primary root shows steady growth. The growth rate of the primary root was calculated as the measured distance of the root apical meristem movement divided by the time interval between the corresponding markings (~24 h). These procedures were completed as described by Kikuchi *et al.* (2023a).

### Kinematic analysis of cells

The root elongation rate at each point using 8-day-old seedlings, which showed steady growth in primary roots, was measured. After the elongation rate was determined, the length of the cortical cells and the inner and outer radius of the cortical cell file were examined using the ImageJ Plugin ‘Root Cell Size Measurer’ (in Ij Tool; LPixel, Tokyo, Japan) as described by Iwamoto *et al.* (2006). The root elongation rate and the length of the cortical cell file were converted to the volume flux rate and cell volume. Additionally, each growth parameter was calculated. These procedures were completed as described by Iwamoto *et al.* (2006).

### Whole-mount fluorescence *in situ* hybridization analysis

The WM-FISH analysis was performed using the whole root region, which was preserved by attaching the roots to MAS-coated glass slides (Matsunami Glass, Osaka, Japan). Centromeres were fluorescently labeled with Cy3-dCTP, whereas nuclei were stained with DAPI. Confocal images were acquired using a confocal laser scanning microscope (FV1000-D; Olympus, Tokyo, Japan) and converted to 3D images using the Amira software (version 2019.4) (Mercury Computer Systems, Berlin, Germany). The nuclear volume and the number of centromere signals in each root region were calculated using Amira’s label analysis module. These procedures were performed as described by Kikuchi *et al.* (2023b). Note that the root samples were not crushed but left intact, keeping their whole structures with this analysis method.

### Numerical calculations for kinematic analysis

The R program (R for Mac OS 4.0.3; R Development Core Team, 2020) was used for calculating each growth parameter of kinematic analysis.

### Statistical analysis

Welch’s *t*-test was conducted with two datasets of the kinematic growth parameters to see if there is a significant difference between the diploid and autopolyploids (i.e. diploid versus tetraploid-1, diploid versus hexaploid-1, diploid versus otctoploid-1; dataset 1; and diploid versus tetraploid-2, diploid versus hexaploid-2, diploid versus otctoploid-2; dataset 2). Note that only the results of Welch’s *t*-test for dataset 1 are shown in the figures, except for in specific regions, because the results of Welch’s *t*-tests for these two datasets were almost same. The results of Welch’s *t*-test for dataset 2 are shown only in the region where the results of two Welch’s *t*-tests were different.

Bartlett tests were conducted for the numbers of centromere signals between growth regions in the diploid and autopolyploids. The results of the analysis indicated homogeneity of variance for the diploid and tetraploid datasets, but not for the hexaploid and octoploid datasets. Therefore, one-way ANOVA on the diploid and tetraploid datasets and Kruskal–Wallis tests on the hexaploid and octoploid datasets were conducted. Since these tests showed a significance difference (*P*<0.01) in all datasets, Tukey HSD post-hoc tests were conducted on the diploid and tetraploid datasets and Steel–Dwass tests on the hexaploid and octoploid datasets.

The Bartlett test and the Steel–Dwass test were conducted using the R program. Welch’s *t*-test and Kruskal–Wallis test were conducted using the software KaleidaGraph version 5.0 (Synergy Software, Pleasanton, CA, USA).

## Results

### Kinematic analysis of the root growth of *A. thaliana* autopolyploids

**Fig. 1.**
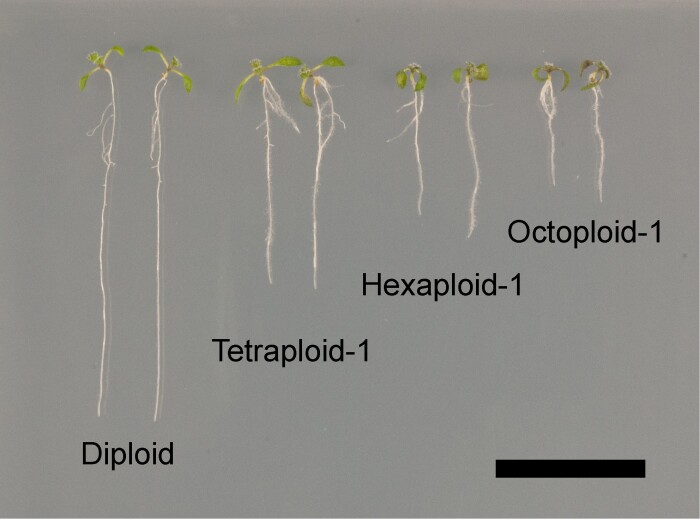
Synthetic autopolyploids (diploid, tetraploid, hexaploid, and octoploid) of *A. thaliana.* Eight-day-old seedlings were grown vertically on solid medium containing MS salts. The length of the primary root was inversely proportional to the ploidy level. The kinematic analysis of cells was performed using the 8-day-old seedlings, which exhibited steady primary root growth. Scale bar=2 cm.

We examined root growth by performing a kinematic analysis of root cortical cells to elucidate the effects of autopolyploidization on *A. thaliana* organ growth. Although root length and growth zones are known to vary from sample to sample, representative growth parameters were calculated by analyzing a sufficient number of samples in this study (diploid, *n*=40; other lines, *n*=30). Each line used in the kinematic analysis is shown in Fig. 1. The root length of 8-day-old seedlings decreased as the ploidy level increased.

**Fig. 2.**
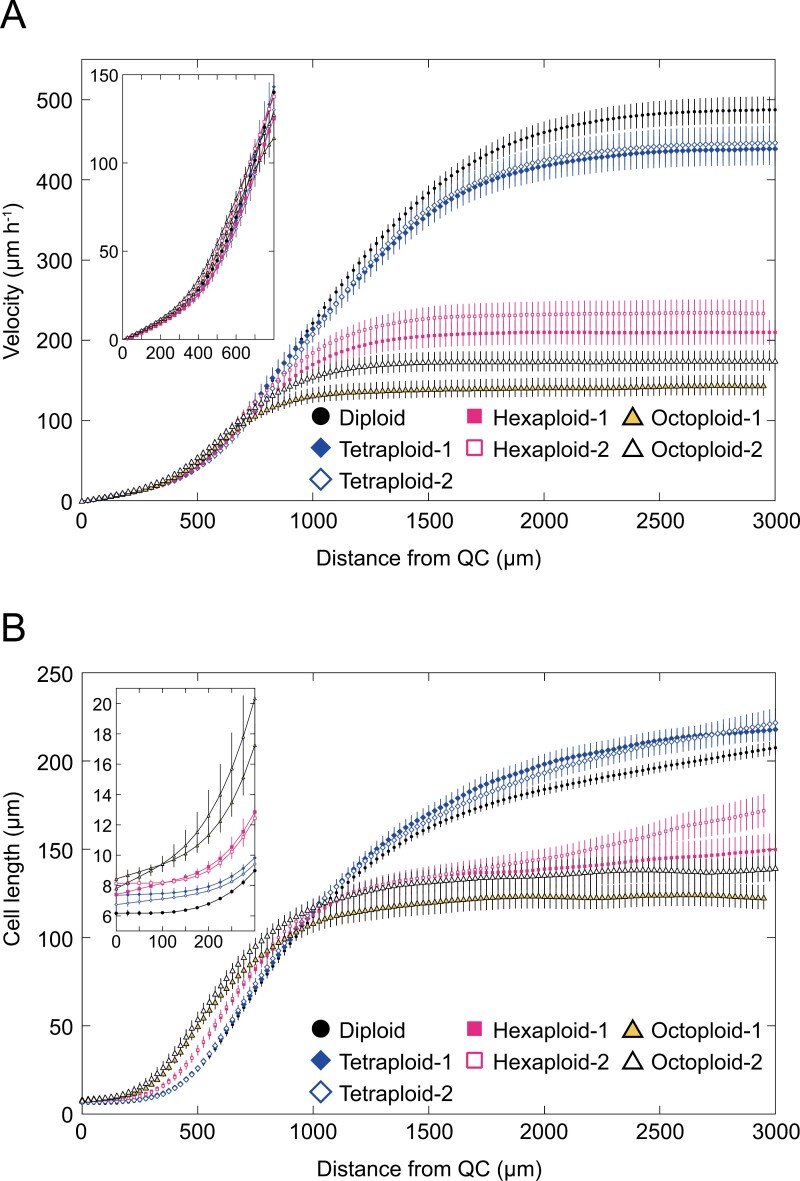
Spatial profiles of the root elongation rate and cell length of *A. thaliana* autopolyploids. Data were plotted versus the distance from the QC. (A) Root elongation rate (velocity). (B) Cell length. Insets present the spatial profiles of the root elongation rate (A) and cell length (B) in the region close to the QC. Bars indicate SEs. Number of seedlings analyzed: *n*=40 (diploid); *n*=30 (other lines).

The kinematic analysis was performed following the method previously described by Iwamoto *et al.* (2006), in which two key parameters, the elongation rate (velocity, Fig. 2A) and cell length (Fig. 2B), were measured, and each growth parameter was calculated based on these. The velocity is the parameter for root elongation rate per hour at any given distance from the quiescent center (QC), and the cell length is the parameter for the longitudinal length of a cell along the root at any given distance from the QC. The velocities were not significantly different among all lines in the distal region of the root, while they decreased inversely proportional to the ploidy level in the proximal region of the root, especially in the high-polyploid lines (Fig. 2A). The cell lengths were larger in the distal region of the root in proportion to the ploidy level, while the cell lengths in the proximal region of the root were significantly smaller in the high-polyploid lines than those in the diploid and tetraploid lines (Fig. 2B).

**Fig. 3.**
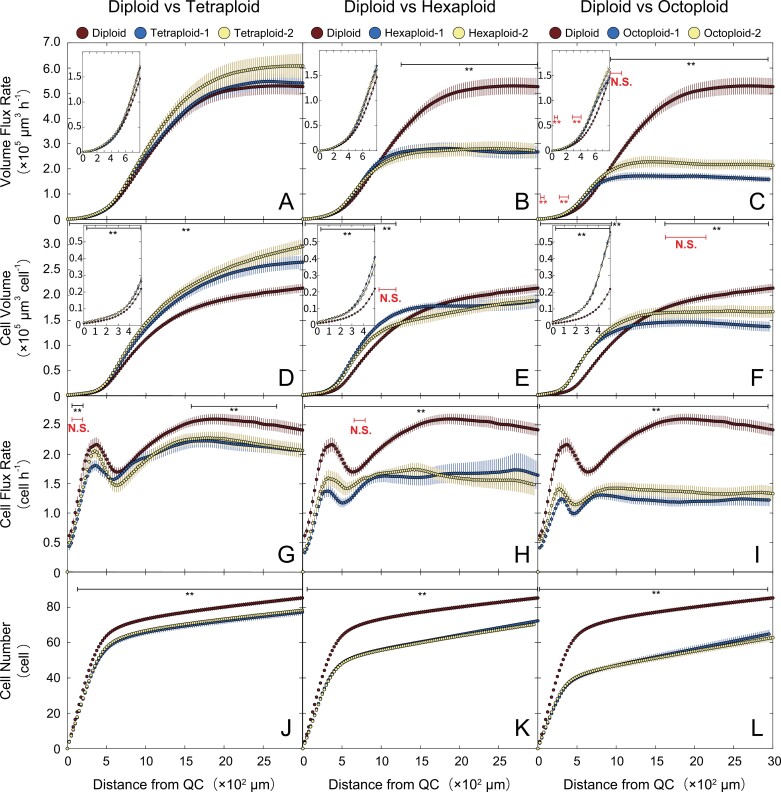
Spatial profiles of the root growth parameters of *A. thaliana* autopolyploids. Data were plotted versus the distance from the QC. (A–C) Volume flux rate. (D–F) Cell volume. (G–I) Cell flux rate. (J–L) Cell number. Insets present the spatial profiles of the volume flux rate (A–C) and cell volume (D–F) in the region close to the QC. Data for the diploid were identical for (A–C), (D–F), (G–I), and (J–L). Bars indicate SEs. Number of seedlings analyzed: *n*=40 (diploid); *n*=30 (other lines). The results of Welch’s *t*-test performed on diploid and tetraploid-1, hexaxploid-1, and octoploid-1 are shown in black. Asterisks (**) indicate significant differences (*P*<0.01). The results of Welch’s *t*-test performed on diploid and tetraploid-2, hexaxploid-2, and octoploid-2 are shown in red only in the regions where the results of two Welch’s *t*-tests were different. See the text for details of the statistical analysis. Asterisks (**) indicate significant differences (*P*<0.01), N.S. indicates no significant differences.

**Fig. 4.**
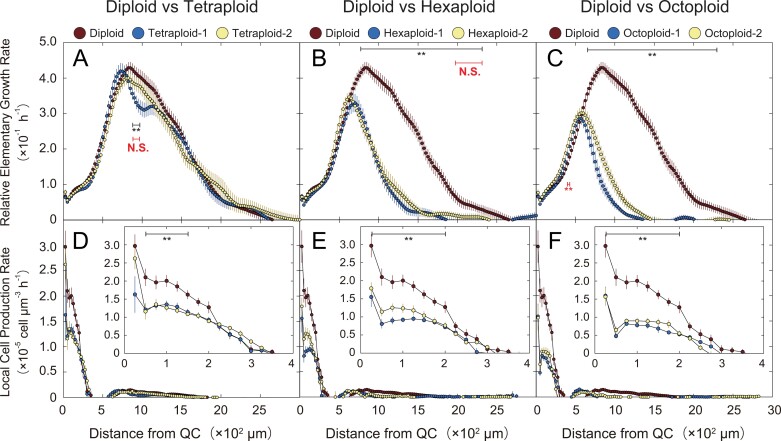
Spatial profiles of REGR and LCPR for the *A. thaliana* autopolyploids. Data were plotted versus the distance from the QC. (A–C) Relative elementary growth rate (REGR). (D–F) Local cell production rate (LCPR). Insets present the spatial profiles of the LCPR in the region close to the QC. Data for the diploid were identical for (A–C) and (D–F). Bars indicate SEs. Number of seedlings analyzed: *n*=40 (diploid); *n*=30 (other lines). The results of Welch’s *t*-test performed on diploid and tetraploid-1, hexaxploid-1, and octoploid-1 are shown in black. Asterisks (**) indicate significant differences (*P*<0.01), N.S. indicate no significant differences. The results of Welch’s *t*-test performed on diploid and tetraploid-2, hexaxploid-2, and octoploid-2 are shown in red only in the regions where the results of two Welch’s *t*-tests were different. See the text for details of the statistical analysis.

We calculated all growth parameters on a volume basis from two key parameters, the velocity and cell length, and the inner and outer radius of the cortical cell file. The calculated growth parameters plotted versus the distance from the QC revealed the spatial profiles of root growth in each line (Figs 3, 4).

We first quantified the volume flux rate (the cumulative cell volume increase per hour in the region from the QC to a given point) and the cell volume (the cell volume at a given point) of each line to analyze the effect of the progression of autopolyploidization on the cell volume increase. The volume flux rates of the tetraploids (tetraploid-1 and tetraploid-2) were almost the same or slightly greater than that of the diploid (Fig. 3A). In contrast, the volume flux rates of the hexaploids (hexaploid-1 and hexaploid-2) and octoploids (octoploid-1 and octoploid-2) were significantly lower than that of the diploid (Fig. 3B, C). The volume flux rates at 2800 μm from the QC (i.e. the point at which the volume flux rates of all lines reached a steady state) were 5.29 × 10^5^ μm^3^ h^−1^ in the diploid, 5.43 × 10^5^ μm^3^ h^−1^ in tetraploid-1, 6.08 × 10^5^ μm^3^ h^−1^ in tetraploid-2, 2.65 × 10^5^ µm^3^ h^−1^ in hexaploid-1, 2.76 × 10^5^ µm^3^ h^−1^ in hexaploid-2, 1.60 × 10^5^ µm^3^ h^−1^ in octoploid-1, and 2.17 × 10^5^ µm^3^ h^−1^ in octoploid-2 (Fig. 3A–C).

The spatial profiles of the cell volume indicated that the tetraploids had larger cortical cells than the diploid throughout the analyzed root region (Fig. 3D). Compared with the corresponding cell volumes of the diploid, the cell volumes of the high polyploids were larger in the distal region of the root up to 1000–1500 µm from the QC, but those of the hexaploids were almost the same and those of the octoploids were even smaller in the proximal region (Fig. 3E, F). In the distal region from the QC to ~700 µm, the cell volume increased according to the ploidy level; setting the cell volume of the diploid as 1.0, the relative cell volume of each line at a given point in this region (0–700 µm) was as follows: tetraploid-1=1.19–1.54, tetraploid-2=1.24–1.56, hexaploid-1=1.54–1.88, hexaploid-2=1.38–1.75, octoploid-1=1.64–2.73, and octoploid-2=1.68–2.70. The cell volume increases occur in the distal region of the root apical meristem in the high polyploids, especially in the octoploids (Fig. 3E, F). The cell volumes at 2800 μm from the QC of each line were 2.10 × 10^5^ µm^3^ in the diploid, 2.63 × 10^5^ µm^3^ in tetraploid-1, 2.89 × 10^5^ µm^3^ in tetraploid-2, 1.85 × 10^5^ µm^3^ in hexaploid-1, 1.87 × 10^5^ µm^3^ in hexaploid-2, 1.39 × 10^5^ µm^3^ in octoploid-1, and 1.67 × 10^5^ µm^3^ in octoploid-2. We also calculated the cumulative cell volume, which was greater in polyploid lines compared with diploids (Supplementary Fig. S1).

We subsequently determined the cell flux rate (the cumulative number of cells produced in the region from the QC to a given point per hour) and cell number (the cumulative cell number in the region from the QC to a given point) to analyze the effect of the progression of autopolyploidization on cell proliferation. The cell flux rate decreased according to the ploidy level throughout the analyzed root region (Fig. 3G–I). The cell flux rates at 2800 μm from the QC were 2.46 cell h^−1^ in the diploid, 2.08 cell h^−1^ in tetraploid-1, 2.10 cell h^−1^ in tetraploid-2, 1.72 cell h^−1^ in hexaploid-1, 1.53 cell h^−1^ in hexaploid-2, 1.23 cell h^−1^ in octoploid-1, and 1.35 cell h^−1^ in octoploid-2. Although in theory the cell flux rate should increase monotonically with distance along the root, it showed a small and localized decrease at ~500 μm from the QC in all lines (Fig. 3G–I). One likely cause for this decrease is the slight underestimation of the volume flux rate in this region, as the cell flux rate was calculated by multiplying the cell density by the volume flux rate (Iwamoto *et al.*, 2006). This issue may arise from technical difficulties in accurately determining the volume flux rate at each position in the region where the volume flux rate rapidly increases. The cell number also decreased according to the ploidy level, which was consistent with the changes in the cell flux rate (Fig. 3J–L). The cell numbers in the region from 25 μm to 2800 μm from the QC were almost the same in lines with the same ploidy level. Compared with the cell number for the diploid, the cell number was 0.84–0.92 times lower for the tetraploids, 0.75–0.85 times lower for the hexaploids, and 0.62–0.81 times lower for the octoploids.

Next, we calculated the relative elementary growth rate (REGR) according to the volume flux rate, as well as the local cell production rate (LCPR) according to the cell flux rate (Fig. 4). The REGR represents the relative rate of the cell volume increase at any given point along the root. The volume growth zone corresponds to the region from the QC to the point where the REGR decreases to 0. In tetraploid-1, REGR peaked at ~725 µm from the QC, whereas in tetraploid-2 and the diploid, it peaked at ~825 µm from the QC. The REGR peak was slightly lower in the tetraploids (tetraploid-1, 4.19 × 10^−1^ h^−1^; tetraploid-2, 4.00 × 10^−1^ h^−1^) than in the diploid (4.29 × 10^−1^ h^−1^). The REGR decreased to 0 at 2650–2675 µm (from the QC) in the diploid, 2600–2625 µm in tetraploid-1, and >3000 µm in tetraploid-2. These results indicate that the polyploidization that generated tetraploids did not expand the volume growth zone (Fig. 4A). However, the polyploidization that generated higher polyploids had a notable effect on the volume growth zone. The REGRs peaked closer to the QC for the hexaploids and octoploids than for the diploid and tetraploids. The REGR peaked at 700 μm (from the QC) in hexaploid-1, 625 μm in hexaploid-2, and 575 μm in octoploid-1 and octoploid-2. The REGR peak was significantly lower for the high polyploids than for the diploid and tetraploids (hexaploid-1, 3.33 × 10^−1^ h^−1^; hexaploid-2, 3.46 × 10^−1^ h^−1^; octoploid-1, 2.86 × 10^−1^ h^−1^; octoploid-2, 3.01 × 10^−1^ h^−1^). The distances from the QC to the point at which the REGR was 0 were 1850–1875 µm in hexaploid-1, 2400–2425 µm in hexaploid-2, 2000–2025 µm in octoploid-1, and 1450–1475 µm in octoploid-2. Thus, the volume growth zone was smaller in the hexaploids and octoploids than in the diploid and tetraploids (Fig. 4A–C).

The LCPR represents the number of cells produced locally per unit time at a given point in the root cortical cell file. The cell proliferation zone (PZ) was defined as the region from the QC to the point where the LCPR decreased to 0. In the region near the QC (<200 µm from the QC), the LCPRs of the autopolyploids decreased almost proportionally to the ploidy level, indicating that the progression of autopolyploidization suppressed cell proliferation (Fig. 4D–F, insets). The size of the PZ was 350–375 µm in the diploid, 325–350 µm in tetraploid-1 and tetraploid-2, 275–300 µm in hexaploid-1, 300–325 µm in hexaploid-2, 275–300 µm in octoploid-1, and 250–275 µm in octoploid-2. Accordingly, the size of the PZ decreased slightly in the autopolyploids. We also plotted the cell flux rate versus the number of cortical cells from the QC (Supplementary Fig. S2A–C) and calculated the LCPR as the derivative of cell flux rate (Supplementary Fig. S2D–F). The number of cortical cells in the PZ decreased according to the ploidy level. Specifically, the cell number in the PZ was 57 in the diploid, 49 in tetraploid-1, 46 in tetraploid-2, 38 in hexaploid-1, 41 in hexaploid-2, 31 in octoploid-1, and 33 in octoploid-2.

### WM-FISH analysis of *A. thaliana* autopolyploids

**Fig. 5.**
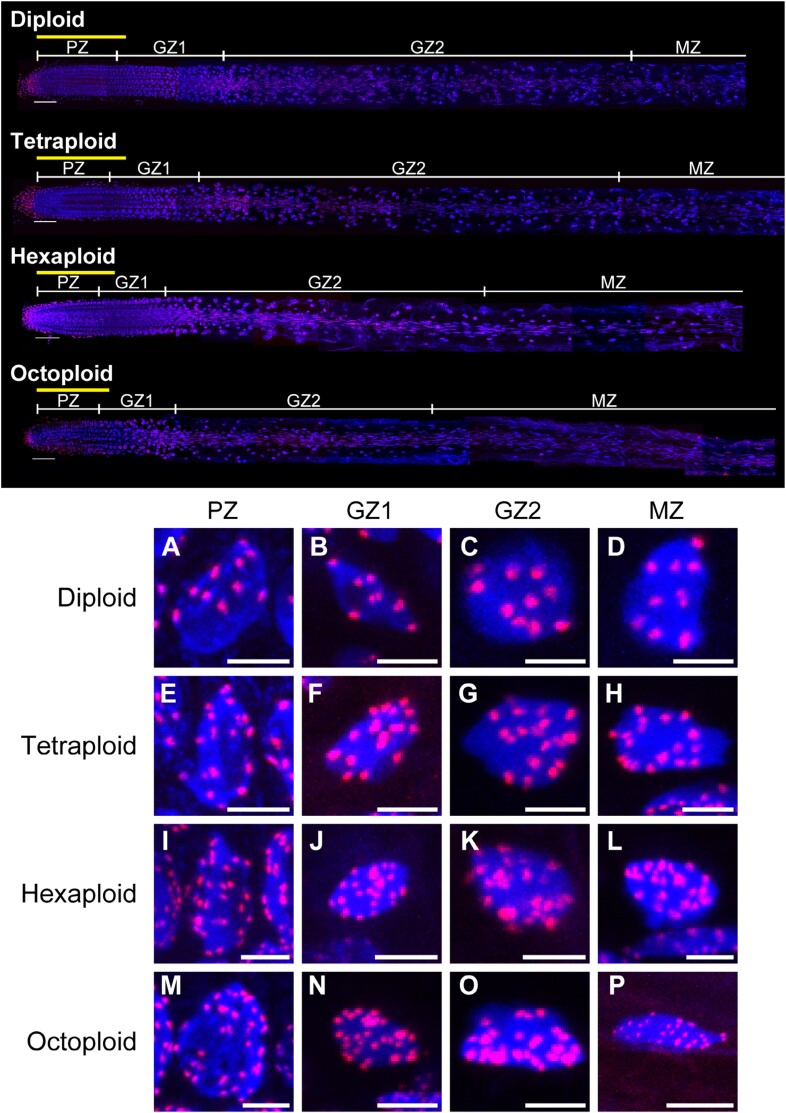
Confocal images of the whole-mount FISH (WM-FISH) analysis. The novel WM-FISH technique successfully detected the fluorescent signals of DAPI (nuclei, blue) and Cy3 (centromere, red) in each region of the intact root of *A. thaliana* autopolyploids. Maximum intensity projection images are shown. The whole root of each line is presented in the upper panel. Yellow bars in the upper panel indicate the meristem size based on conventional classification by cell size. Magnified views of the nucleus in each growth region of the root of each line (PZ, cell proliferation zone; GZ1, growth zone-1; GZ2, growth zone-2; MZ, mature zone) are presented in the lower panels (A–P). Scale bars=100 µm (upper panel, ×40 magnification), 5 µm (A, E, I, and M, ×100 magnification), 10 µm (B–D, F–H, J–L, N, and O, ×40 magnification), and 20 µm (P, ×40 magnification).

We performed the WM-FISH analysis using the whole roots of *A. thaliana* plants to detect the changes in chromosome behavior due to polyploidization. The WM-FISH analysis method described by Kikuchi *et al.* (2023b) was applicable to the autopolyploid series (Fig. 5). On the basis of the results of the root growth analysis (Fig. 4), WM-FISH data were obtained for the following four growth regions: the region between the QC and the point where the LCPR decreased to 0 (i.e. PZ); the region between the point where the LCPR decreased to 0 and the point where the REGR peaked, which was defined as growth zone-1 (GZ1); the region between the point where the REGR peaked and the point where the REGR decreased to 0, which was defined as growth zone-2 (GZ2); and the region beyond the point where the REGR decreased to 0, which was defined as the mature zone (MZ) (Table 1). It should be noted that GZ1 includes a few meristematic cells in actual roots, as this region lies between the PZ and the non-cell proliferation zone. In other words, the region corresponds to the transition domain of the root apical meristem. Therefore, we included part of GZ1 in the root apical meristem (Fig. 5, upper panel).

**Table 1.** Range of each growth zone in autopolyploid series of *A. thaliana*.

	Cell proliferation zone (PZ)	Growth zone-1 (GZ1)	Growth zone-2 (GZ2)	Mature zone (MZ)
Diploid	QC<PZ≦350 µm	350<GZ1≦825 µm	825<GZ2≦2650 µm	2650 µm<MZ
Tetraploid	QC<PZ≦325 µm	325<GZ1≦725 µm	725<GZ2≦2600 µm	2600 µm <MZ
Hexaploid	QC<PZ≦275 µm	275<GZ1≦700 µm	700<GZ2≦1850 µm	1850 µm<MZ
Octoploid	QC<PZ≦275 µm	275<GZ1≦575 µm	575<GZ2≦2000 µm	2000 µm<MZ

Each growth region was defined by the relative elementary growth rate and the local cell production rate calculated by the kinematic analysis (see the text for details).

**Fig. 6.**
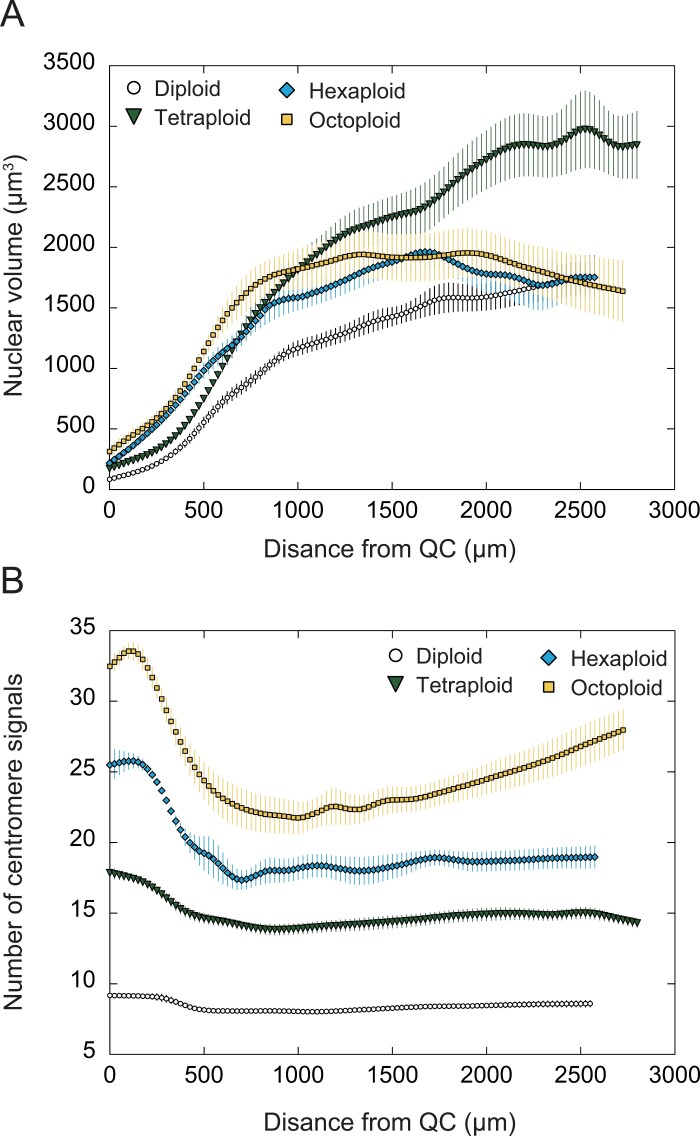
Whole-mount FISH (WM-FISH) analysis of the root apical meristem of *A. thaliana* autopolyploids. Data were plotted versus the distance from the QC. (A) Nuclear volume. (B) Number of centromere signals. Bars indicate SEs. Number of seedlings analyzed: *n*=10 (each line). Graphs present the fitted curves obtained by smoothing (Friedman super smoother) and interpolating (spline interpolation) the raw data of the number of centromere signals and nuclear volume, respectively.

The spatial pattern of the nuclear volume was similar between the diploid and tetraploids, with a gradual increase from the apical region to the basal region (Fig. 6A). Overall, the nuclear volume was greater for the tetraploid than for the diploid; in the MZ, the nuclei of the tetraploid were 1.73–1.82 times larger than those of the diploid. The spatial pattern of the nuclear volume of the high polyploids (hexaploid and octoploid) differed from that of the diploid and tetraploid; the nuclear volume almost reached a plateau in GZ2 and MZ (Fig. 6A; Table 1). In terms of the whole root, the hexaploid and octoploid had almost the same nuclear volume spatial profile, whereas the octoploid had a slightly larger nuclear volume than the hexaploid in the region ~500–1500 µm from the QC. Although the basic nuclear DNA content was higher in the hexaploid and octoploid than in the diploid, the nuclear volumes in the MZ were similar (i.e. only 1.04–1.15 times greater in the hexaploid and 0.94–1.15 times greater in the octoploid than in the diploid).

We also calculated the ratio of nuclear volume to cell volume, as determined by kinematic analysis and WM-FISH analysis, respectively, and plotted it as a function of distance from the QC (Supplementary Fig. S3). In all lines, the ratio of nuclear volume to cell volume was high in the distal region of the root apical meristem, while the ratio decreased sharply in the cell volume growth zone and became constant in the MZ.

**Fig. 7.**
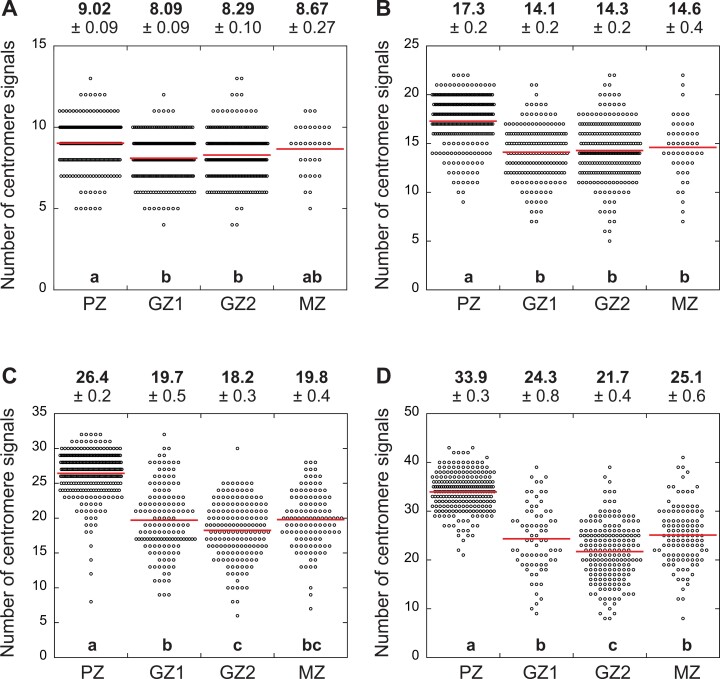
One-dimensional scatter plot of the number of centromere signals in the nucleus of each line. (A) Diploid: *n*=245 (PZ, cell proliferation zone), 248 (GZ1, growth zone-1), 238 (GZ2, growth zone-2), and 33 (MZ, mature zone). (B) Tetraploid: *n*=261 (PZ), 178 (GZ1), 234 (GZ2), and 50 (MZ). (C) Hexaploid: *n*=304 (PZ), 144 (GZ1), 164 (GZ2), and 119 (MZ). (D) Octoploid: *n*=237 (PZ), 73 (GZ1), 194 (GZ2), and 118 (MZ). Red lines represent the mean. The number of centromere signals in each region of each line (mean ±SE) is indicated above each graph. Different lowercase letters in each graph indicate significant differences (*P*<0.05) (A, B: Tukey HSD post-hoc test; C, D: Steel–Dwass test).

The number of centromere signals can be used to estimate the degree of chromosome polytenization because the number of centromere signals reportedly decreases in nuclei with polytenized chromosomes (Breuer *et al.*, 2007; Kikuchi and Iwamoto, 2020). The diploid and tetraploid had similar spatial patterns in the number of centromere signals, which was nearly constant in the whole root, although it was slightly higher in the PZ than in GZ1, GZ2, and the MZ (Figs 6B, 7A, B; Table 1). In the hexaploid and octoploid, the number of centromere signals was much lower in GZ1, GZ2, and the MZ than in the PZ (Figs 6B, 7C, D; Table 1). This result reflected the severe chromosome polytenization in the hexaploid and octoploid. Interestingly, chromosome polytenization was more extensive in GZ2 than in GZ1 in the hexaploid and octoploid, which distinguishes them from the diploid and tetraploid.

## Discussion

### Progression of polyploidization suppresses cell proliferation

The root growth analysis showed that the LCPR decreased in the PZ as the polyploidization progressed (Fig. 4D–F). Because the LCPR represents the number of cells produced at a specific location per hour, our data indicate that polyploids, especially high polyploids, have a longer cell cycle than diploids. These results are consistent with those of previous studies that revealed the strong positive correlation between the nuclear DNA content and the duration of the cell cycle (Hof and Sparrow, 1963; Bennett *et al.*, 1972; Francis *et al.*, 2008). Earlier research confirmed that the cell cycle duration is primarily determined by the S phase (Francis *et al.*, 2008; Šímová and Herben, 2012). The duration of the S phase will be constrained by the total amount of DNA and the transport rate of the components required for DNA replication (e.g. DNA polymerase) (Šímová and Herben, 2012). Such constraints may also occur in polyploids, which have more nuclear DNA than diploids.

The graph plotting the LCPR against the cell number from the QC showed that the number of cells in the PZ decreased significantly as the ploidy level increased (Supplementary Fig. S2D–F). This result suggests that the maximum number of cell proliferations for a cortical cell decreases according to the ploidy level.

### Tetraploidization increases the cell volume, whereas further polyploidizations have the opposite effect

The findings of the root growth analysis showed that the increase in cell volume in the steady-state growth region due to polyploidization occurred in tetraploids, but not in the high polyploids (Fig. 3D–F). However, the spatial pattern of the REGR was almost the same for the diploid and tetraploids (Fig. 4A), suggesting that the cortical cell file of the tetraploids probably has fewer cells than the corresponding region of the diploid and that the growth rate of each cell is higher in the tetraploids than in the diploid. The volume growth zone and the REGR peak were significantly smaller in the high polyploids than in the diploid and tetraploids (Fig. 4A–C), indicating that the cell volume increase in the high polyploids was severely suppressed in the volume growth zone of the root. The decrease in the cell volume and volume increases due to polyploidization may be caused by several physical factors, among which the most likely factor is the change in the cell wall composition of high polyploids. As polyploidization progresses, the cell wall thickness decreases in the stem and the vascular bundle tissue becomes abnormal. More specifically, octoploids have a significantly thinner cell wall than diploids, tetraploids, and hexaploids, and they also have a relatively high percentage of abnormal vessels in their vascular tissue (Corneillie *et al.*, 2019). The physical changes (e.g. decreased thickness) to the cell wall of high polyploids may be one of the main factors involved in the decrease in the cell volume and volume flux rate in high polyploids. Corneillie *et al.* (2019) also reported that the cell wall composition changes in the stem of high polyploids, which may also influence the cell volume and volume flux rate. The thickness of the cell wall and the cell wall composition in the roots of polyploids should be more comprehensively examined in future studies to confirm this hypothesis.

As discussed in the previous section, the polyploids underwent fewer cell divisions than the diploid, but their cell volumes increased in the PZ. Additionally, the increased nuclear DNA content of the tetraploids resulted in increased cell volumes in the volume growth zone, which was in contrast to the suppressed cell volume increase in the hexaploids and octoploids. These results imply that the mechanism that suppresses cell volume increase counteracts the beneficial effects of the increased genomic content due to polyploidization in high polyploids.

### Relationships among the nuclear volume, endoreduplication, and cell volume increase

An earlier fluorescence-activated nuclear sorting analysis showed that there are 2C, 4C, 8C, and 16C cells in the root cortical cell files in the diploid *A. thaliana* (Bhosale *et al.*, 2018). Thus, the MZ of the diploid contained 16C cells and the cortical cells of the diploid underwent at most three rounds of endoreduplication (i.e. 2C to 4C to 8C to 16C; Fig. 6A). Because the DNA content of nuclei is proportional to the nuclear volume (Jovtchev *et al.*, 2006), the MZ of the tetraploid mainly consisted of 32C cells, which was reflected by the nuclear volume. Hence, the cortical cells of the tetraploid also underwent three rounds of endoreduplication (i.e. 4C to 8C to 16C to 32C). These results suggest that the DNA content in the tetraploid was doubled via genomic changes, including endoreduplication. The DNA content is considered to be proportional to the cell volume (Šímová and Herben, 2012). Indeed, the nuclear volume in the MZ was approximately twice as large in the tetraploid than in the diploid (Fig. 6A). Therefore, the larger cell volume and higher volume flux rate in the tetraploid than in the diploid should be attributed to the increased DNA content due to polyploidization as well as endoreduplication (Fig. 3A).

Although the nuclear volumes in the PZ increased almost proportionally to the level of polyploidization in the hexaploids and octoploids (compared with the diploid), the nuclear volumes in the MZ were nearly the same in the hexaploids, octoploids, and diploid (Fig. 6A; Table 1). Accordingly, most of the cortical cells in the hexaploids and octoploids underwent only one round of endoreduplication (i.e. 6C to 12C in hexaploids and 8C to 16C in octoploids); that is, the progression of endoreduplication is likely to be suppressed in high polyploids (relative to the corresponding progression in diploids and tetraploids). The limited increase in the nuclear DNA content resulting from the suppression of endoreduplication can partly explain the suppressed cell volume increase in the high polyploids. For example, the nuclear volume of the high polyploids at 2500 µm from the QC was almost the same as that of the diploid (1.00-fold for hexaploid-1 and 0.97-fold for octoploid-1 relative to the diploid value) (Fig. 6A). If the cell volume simply reflects the nuclear volume (DNA content), then the cells of the diploid and high polyploids at 2500 µm from the QC should have the same volume. However, according to the kinematic analysis, the cell volume at 2500 μm from the QC was significantly smaller for the high polyploids than for the diploid (0.88-fold for hexaploid-1 and 0.69-fold for octoploid-1 relative to the diploid value) (Fig. 3E, F). Therefore, there is likely to be an additional factor suppressing the cell volume increase in high polyploids.

We also calculated the ratio of nuclear volume to cell volume in each growth zone of the root to elucidate the relationship between the nuclear size and cell volume, and found that the regions occupied by nuclei sharply decreased along the root, and the ratio was constant in the MZ (Supplementary Fig. S3). There is no clear correlation between the ratio and cell volume.

### Chromosome polytenization suppresses cell volume increase, but not cell proliferation, in high polyploids

An artificial decrease in the number of centromere signals could occur due to accidental contact between centromeres (Lermontova *et al.*, 2006; Kikuchi and Iwamoto, 2020; Kikuchi *et al.*, 2023b). The number of centromere signals in the GZ1, GZ2, and MZ of the high polyploids, however, was significantly smaller than that in the PZ, and the decrease of centromere signals in the GZ1, GZ2, and MZ was more profound in the high polyploids than in the diploid (Figs 6B, 7). Therefore, we concluded that the decrease of centromere signals in high polyploids cannot be explained only by artifact and that the chromosome polytenization in the high polyploids contributes to the decreased REGR in GZ2 and the decreased cell volume in the MZ of the high polyploids (Figs 3E, F, 4B, C), which indicates that the chromosome polytenization suppresses cell volume increase, but not cell proliferation in the high polyploids.

The mechanism of the chromosome polytenization is still unknown, but future studies focusing on proteins involved in chromosome adhesion and contact, such as cohesin (Nasmyth and Haering, 2009), in the high polyploids may shed light on the mechanism. We will also examine the chromatin accessibility and DNA interactions in the high polyploids using ATAC-seq and Hi-C seq analyses, which, together with the chromosome polytenization, may provide a molecular basis for the high-ploidy syndrome (Agbleke *et al.*, 2020).

**Fig. 8.**
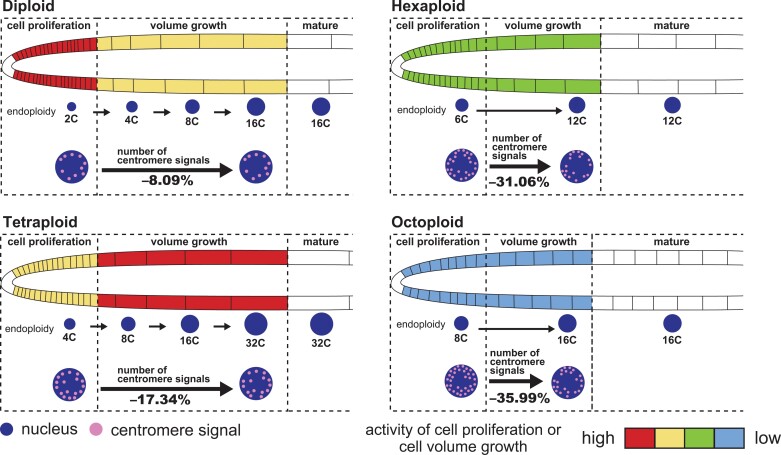
Relationship between growth changes and chromosome polytenization due to autopolyploidization in the *A. thaliana* root.

In this study, we revealed the potential relationship between growth changes and chromosome polytenization due to autopolyploidization by combining the results of the kinematic analysis at the cellular level and the WM-FISH analysis (Fig. 8). Our findings indicate that clarifying the effects of chromosome dynamics will help elucidate the mechanisms mediating growth changes, especially those associated with high-ploidy syndrome. The progression of chromosome polytenization in the high polyploids, estimated by our WM-FISH, will provide for future studies to elucidate the degree of chromosome polytenization in more detail. Future studies should focus on how autopolyploidization alters chromatin structure as well as the changes in gene expression levels associated with chromatin structural changes to further characterize the effects of autopolyploidization.

## Supplementary data

The following supplementary data are available at *JXB* online.

Fig. S1. The results of the kinematic analysis: the cumulative cell volume plotted versus the distance from the QC.

Fig. S2. The results of the kinematic analysis: the cell flux rates and local cell production rates plotted versus the cell number from the quiescent center (QC).

Fig. S3. The ratio of nuclear volume to cell volume plotted versus the distance from the QC.

erae288_suppl_Supplementary_Figures_S1-S3

## Data Availability

All relevant data are available from the authors on request.

## Abbreviations:

GZ1: , growth zone-1;
GZ2: , growth zone-2
LCPR: local cell production rate
MZ: mature zone
PZ: cell proliferation zone
QC: quiescent center
REGR: relative elementary growth rate.
