# Supplementary material for: Plant chromosome polytenization contributes to suppression of root growth in high polyploids
Source: J Exp Bot. 2024 Jul 5;75(18):5703–16. doi: 10.1093/jxb/erae288 (PMC11538578; doi:10.1093/jxb/erae288)
Supplement: erae288_suppl_Supplementary_Figures_S1-S3 [file erae288_suppl_supplementary_figures_s1-s3.pdf]

Title: Plant chromosome polytenization contributes to suppression of the root growth in high-polyploids
Authors: Suzuka Kikuchi, Takuya Sakamoto, Sachihiro Matsunaga, Munetaka Sugiyama, Akitoshi Iwamoto

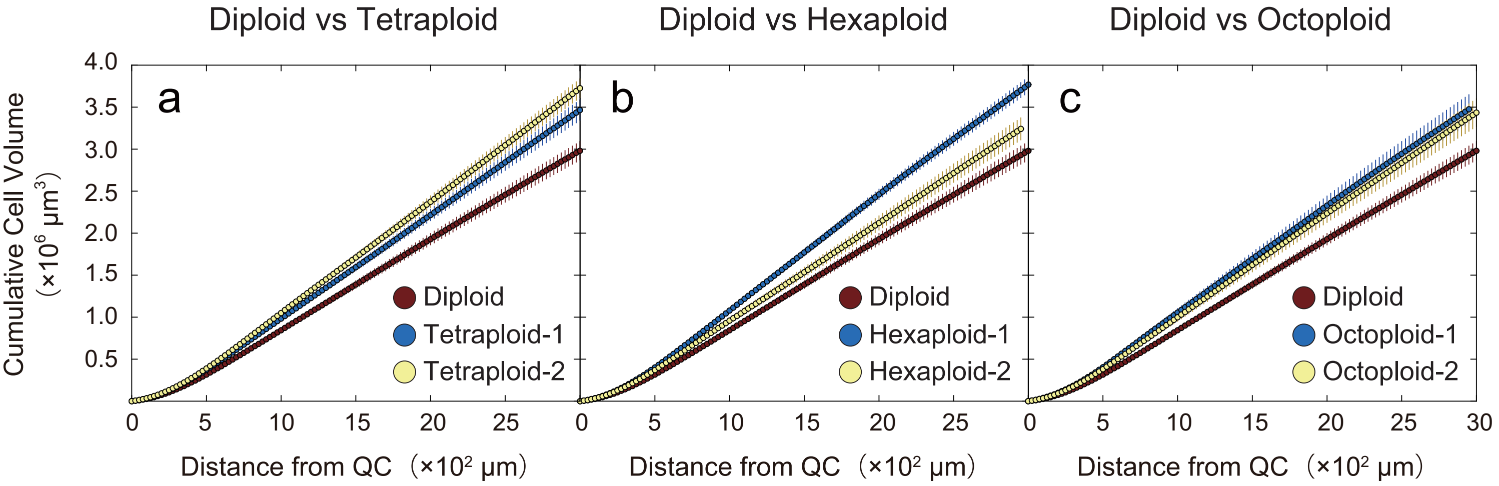

Supplemental Figure 1. Spatial profiles of the cumulative cell volume for the *A. thaliana* autopolyploids Data were plotted versus the distance from the QC. Data for the diploid were identical for (a–c). Bars indicate standard errors. Number of seedlings analyzed: n = 40 (diploid); n = 30 (other strains)

Title: Plant chromosome polytenization contributes to suppression of the root growth in high-polyploids
Authors: Suzuka Kikuchi, Takuya Sakamoto, Sachihiro Matsunaga, Munetaka Sugiyama, Akitoshi Iwamoto

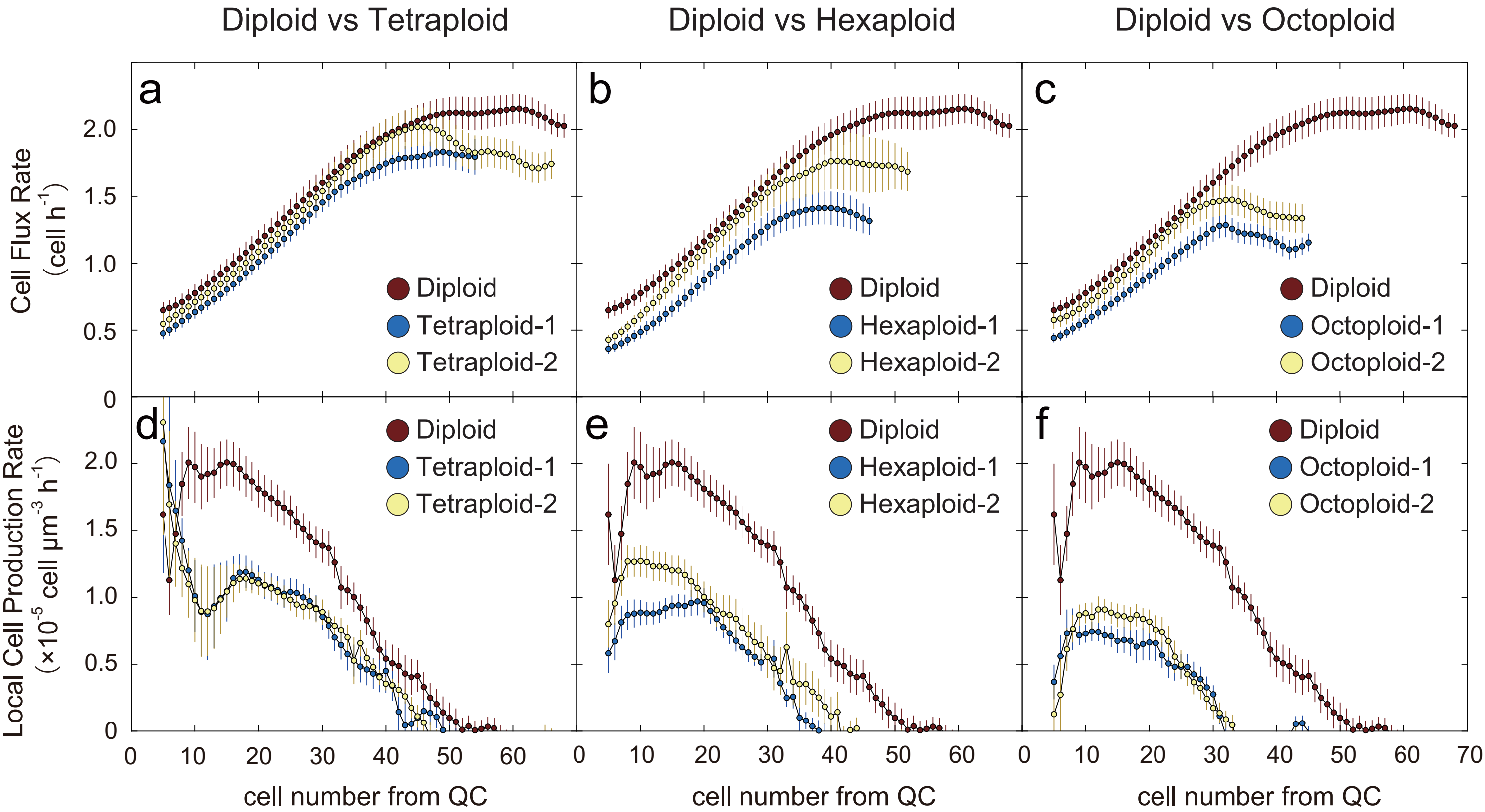

Supplemental Figure 2. Spatial profiles of the cell flux rate and the local cell production rate (LCPR) for the *A. thaliana* autopolyploids
Data were plotted versus the cell number from the QC. (a–c) Cell flux rate. (d–f) Local cell production rate (LCPR). Data for the diploid were identical for (a–c) and (d–f). Bars indicate standard errors. Number of seedlings analyzed: n = 40 (diploid); n = 30 (other strains)

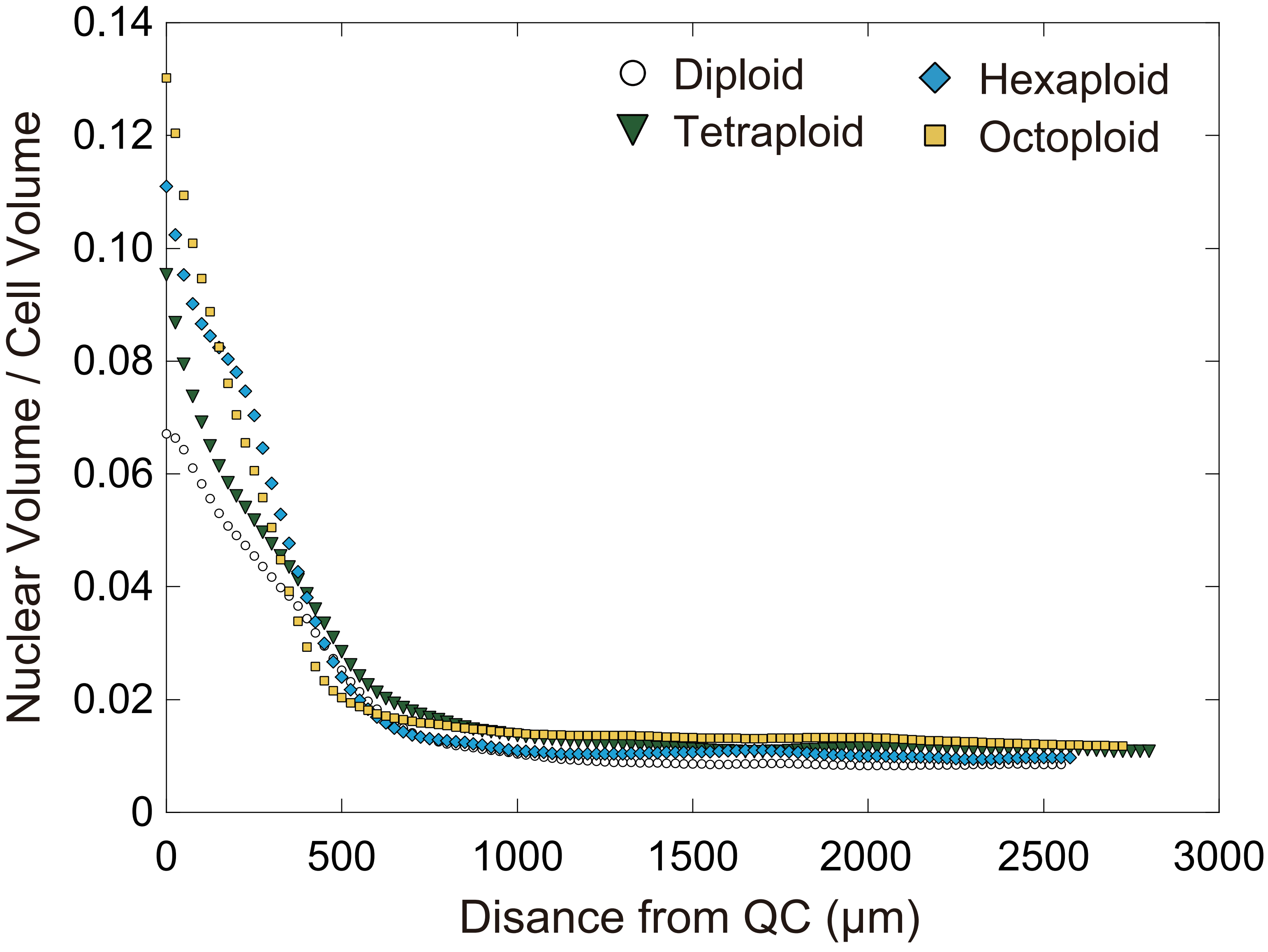

Supplemental Figure 3. Relationship between nuclear volume and cell volume in diploid and each polyploid line
The vertical axis indicates the ratio of nuclear volume to cell volume. Data were plotted versus the distance from the QC. Number of seedlings analyzed: n = 40 (diploid); n = 30 (other strains).
